# Progress in Carbon Nanostructures: From Synthesis to Applications

**DOI:** 10.3390/nano13152181

**Published:** 2023-07-26

**Authors:** Marianna V. Kharlamova, Christian Kramberger, Alexander I. Chernov

**Affiliations:** 1 Faculty of Physics, University of Vienna, Strudlhofgasse 4, 1090 Vienna, Austria; christian.kramberger-kaplan@univie.ac.at; 2 Phystech School of Biological, and Medical Physics, Moscow Institute of Physics and Technology, National Research University, 9 Institutskiy per., 141701 Dolgoprudny, Russia; 3 Russian Quantum Center, Skolkovo Innovation City, 30 Bolshoy Bulvar, 121205 Moscow, Russia; ach@rqc.ru; 4 Center for Photonics and 2D Materials, Moscow Institute of Physics and Technology, National Research University, 9 Institutskiy per., 141701 Dolgoprudny, Russia

Significant progress in carbon nanostructures has been achieved in the past 20 years; however, there is plenty of room for further study. Researchers must bring developments from the laboratory to an industrial scale. The interest in carbon nanostructures is ever growing. Carbon nanotubes, graphene, graphene nanoribbons, 2D heterostructures, fullerenes, nanodiamonds, filled carbon nanotubes (CNTs), and related carbon nanostructures should be realized in applications. On the fundamental side, topics such as synthesis and growth methods, as well as modification of properties, have been considered. Theoretical studies for modeling properties have also been reported. In experimental materials science, the chemical and physical properties of new carbon nanostructures are considered to be promising. The kinetics of the growth of carbon nanostructures is attractive for fundamental and applied research. Activation energy and growth rates inside metallocene-filled carbon nanotubes have been measured for applications. On the applied side, four spectroscopic methods have been implemented on carbon nanostructures to study the kinetics and electronic properties of materials in depth. Among them are Raman spectroscopy, near-edge X-ray absorption fine structure spectroscopy, photoemission spectroscopy, and optical absorption spectroscopy. Applications of new carbon nanostructures include molecular electronics, thermoelectric power generation, light emission, construction materials, and medicine.

In this Special Issue, entitled “Progress in Carbon Nanostructures: From Synthesis to Applications”, we have published four papers, including two review papers [1,2,3,4]. 

In Ref. [1], M. Kharlamova considered issues of the kinetics of growth of filled single-walled carbon nanotubes (SWCNTs) and their electronic properties. Spectroscopic data on carbon nanotubes were discussed. The kinetics included the calculations of growth rates and activation energies of SWCNTs inside SWCNTs encapsulating metallocene molecules. The highlighted spectroscopic methods are Raman spectroscopy, near-edge X-ray absorption fine-structure spectroscopy (NEXAFS), photoemission spectroscopy (PES), and optical absorption spectroscopy (OAS) (Figure 1). Metal halogenides and metal chalcogenides result in n- or p-doping of SWCNTs [5,6,7,8,9,10,11,12,13,14,15,16,17,18]. In this review, the correlations between the chemical nature of the compound and its electronic properties are summarized. They are related to the work function differences between the pristine carbon nanotubes and the compounds.

In Ref. [2], the issues of the cytotoxicity of carbon nanotubes, graphene, fullerene, and dots were considered. The materials characterizations and theoretical considerations are covered. The data of scanning electron microscopy (SEM), transmission electron microscopy (TEM), Raman spectroscopy, OAS, fluorescence spectroscopy, and Fourier transform infrared spectroscopy are discussed. Cell viability and drug release issues are highlighted (Figure 2), and bioimaging issues of carbon nanomaterials are described. As a perspective, the single-cell viability of carbon nanotubes is discussed. Cancer prevention in single cells is needed. This stimulates the development of single-cell methods of analysis, such as microscopy and spectroscopy. Further advancements in drug loading and bioimaging are needed to lower the cytotoxicity. 

In Ref. [3], the authors developed a new method for directly growing patterned vertical graphene on a SiO_2_/Si substrate by plasma-enhanced chemical vapor deposition (PECVD) with patterned Cr film. The quality of the grown vertical graphene was investigated by Raman spectroscopy (Figure 3). The Raman spectrum of graphene includes the characteristic D, G, and 2D modes. Mapping results for D, G, 2D, and ratios D/G and 2D/G are presented. In Figure 4, the schematic of the patterned vertical graphene growth mechanism is presented. The steps are before growth (Figure 4a), heating (Figure 4b), reaching a maximum (σ_max_ = −660 MPa) compressive stress σ_max_ in the Cr film (Figure 4c), growth (Figure 4d), cooling (Figure 4e), and decreasing temperature to T_y_, where vertical graphene/Cr cracks and warps (Figure 4f). This method is very promising, and it proves the possibility of growing graphene on Cr films. 

We acknowledge all authors for their contributions. Please submit your original articles and review papers to the Special Issue “Advanced Carbon Nanostructures: Synthesis, Properties, and Applications II.” 

## Figures and Tables

**Figure 1:**
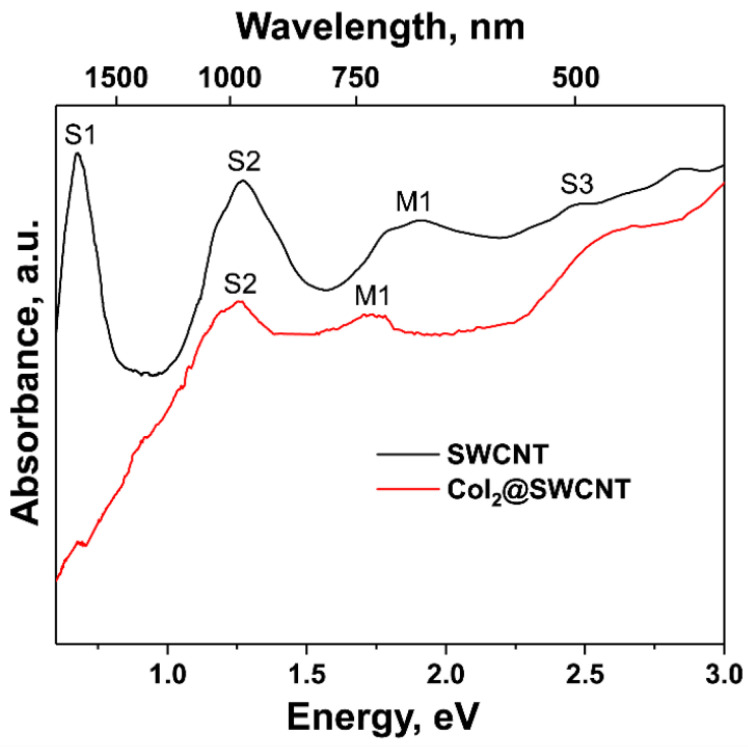
OAS spectra of cobalt iodide-filled SWCNTs [1]. Copyright 2023 by the authors. Licensee: MDPI, Basel, Switzerland. This article is an open-access article distributed under the terms and conditions of the Creative Commons Attribution (CC BY) license.

**Figure 2:**
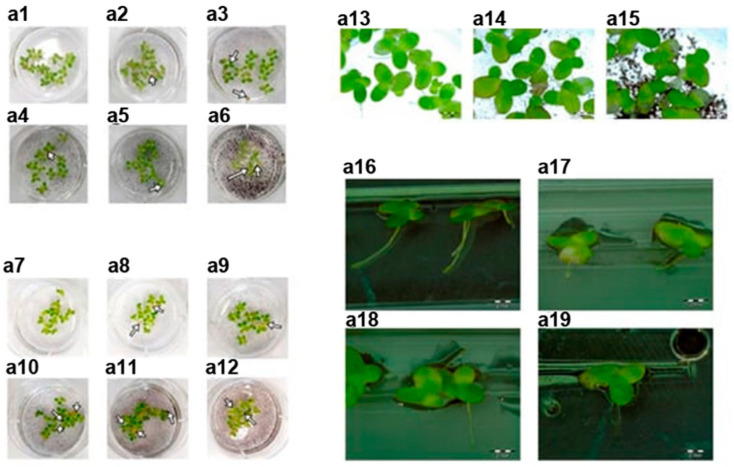
Lemna minor after treatment of raw graphene and graphene oxide (GO). Control group: (**a1**–**a6**,**a13**,**a14**,**a16**,**a17**) after treatment of pristine graphene; (**a7**–**a12**,**a15**,**a18**,**a19**) after GO treatment. Copyright 2021 by the authors. Licensee: MDPI, Basel, Switzerland. This article is an open-access article distributed under the terms and conditions of the Creative Commons Attribution (CC BY) license [19].

**Figure 3:**
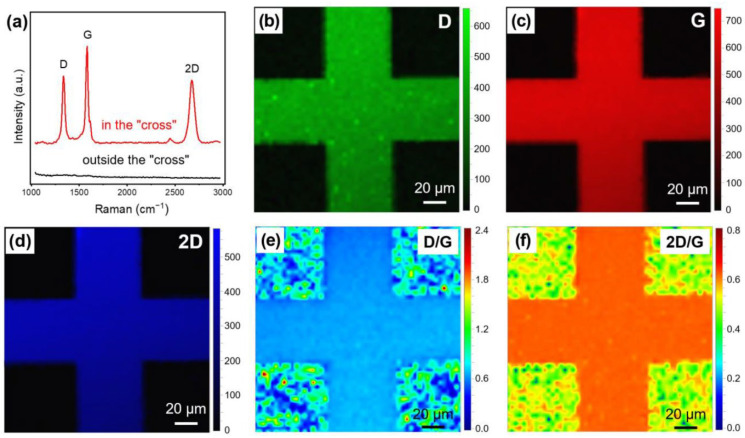
(**a**) Raman spectra of random points on and outside the patterned vertical graphene area. Mapping results for D (**b**), G (**c**), 2D (**d**), and ratios D/G (**e**) and 2D/G (**f**). The mapping area is 150 μm × 150 μm, and 2601 data points were used. Copyright 2023 by the authors. Licensee: MDPI, Basel, Switzerland. This article is an open-access article distributed under the terms and conditions of the Creative Commons Attribution (CC BY) license [3].

**Figure 4:**
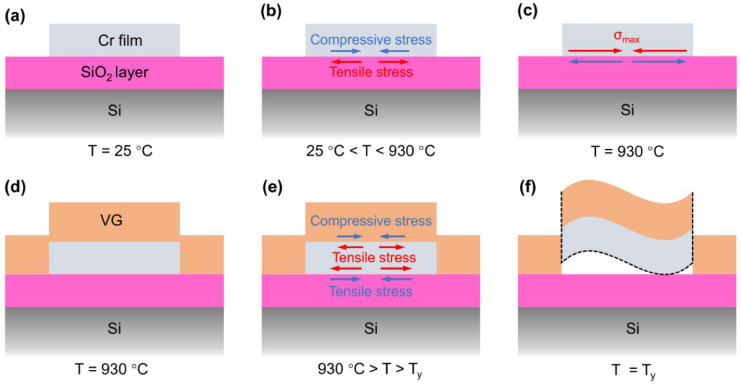
Schematic of the patterned vertical graphene growth mechanism. The steps are before growth (**a**), heating (**b**), reaching a maximum (σmax = −660 MPa) compressive stress in the Cr film (**c**), growth (**d**), cooling (**e**), and decreasing temperature to Ty, where vertical graphene/Cr cracks and warps (**f**). Copyright 2023 by the authors. Licensee: MDPI, Basel, Switzerland. This article is an open-access article distributed under the terms and conditions of the Creative Commons Attribution (CC BY) license [3].
